# An Integrated Multi-Omics Approach Reveals the Effects of Supplementing Grass or Grass Hay with Vitamin E on the Rumen Microbiome and Its Function

**DOI:** 10.3389/fmicb.2016.00905

**Published:** 2016-06-10

**Authors:** Alejandro Belanche, Alison H. Kingston-Smith, Charles J. Newbold

**Affiliations:** Institute of Biological, Environmental and Rural Sciences, Aberystwyth UniversityAberystwyth, UK

**Keywords:** grass, hay, methanogenesis, rumen fermentation, rumen microbiome, Rusitec, vitamin E

## Abstract

Rumen function is generally suboptimal leading to losses in methane and nitrogen. Analysis of the rumen microbiome is thus important to understanding the underlying microbial activity under different feeding strategies. This study investigated the effect of forage conservation method and vitamin E supplementation on rumen function using a rumen simulation technique. Ryegrass (GRA) or ryegrass hay (HAY) was supplemented with 20% concentrate containing zero or 50 IU/d vitamin E, as α-tocopheryl acetate, according to a 2 × 2 factorial design. The forage conservation method did not substantially change the nutrient composition but had a profound impact on the structure and diversity of the rumen microbiome. HAY diets promoted a more complex bacterial community (+38 OTUs) dominated by *Firmicutes*. This bacterial adaptation, together with increased rumen protozoa levels and methanogen diversity, was associated with greater fiber disappearance (+12%) in HAY diets, but also with greater rumen true N degradability (+7%) than GRA diets. HAY diets also had a higher metabolic H recovery and methane production (+35%) suggesting more efficient inter-species H transfer between bacteria, protozoa and methanogens. Contrarily, GRA diets promoted more simplified methanogen and bacterial communities, which were dominated by *Bacteroidetes* and *Lactobacillus*, thus lactate formation may have acted as an alternative H sink in GRA diets. Moreover the structure of the bacterial community with GRA diets was highly correlated with N utilization, and GRA diets promoted greater bacterial growth and microbial protein synthesis (+16%), as well as a more efficient microbial protein synthesis (+22%). A dose-response experiment using batch cultures revealed that vitamin E supplementation increased rumen fermentation in terms of total VFA and gas production, with protozoal activity higher when supplying α-tocopheryl acetate vs. α-tocopherol. Moreover, α-tocopheryl acetate promoted a small increase in feed degradability (+8%), possibly as a result of its antioxidant properties which led to higher bacterial and protozoal levels. Vitamin E supplementation also modified the levels of some methanogen species indicating that they may be particularly sensitive to oxidative stresses. Our findings suggested that when possible, grass should be fed instead of grass hay, in order to improve rumen function and to decrease the environmental impact of livestock agriculture.

## Introduction

The rumen represents a complex microbial ecosystem which enables ruminants to efficiently utilize forages; as a result ruminants are the only livestock which potentially do not compete for human edible foods (Gill et al., 2010). Fresh grass has traditionally been a major feedstuff for ruminants with preserved forages, such as hay and silage, being used when the fresh grass is unavailable. It has been observed that grazing systems generally have a positive impact on animal health and welfare (Somers et al., 2005), milk fatty acid profile (Mohammed et al., 2009) and farm profitability (Kennedy et al., 2005). Moreover, grazing systems also have a positive perception in society as they contribute to maintaining the landscape and animal and plant biodiversity (Fahrig et al., 2011).

Modern, large-scale farms with high yielding dairy cattle or feedlot beef production tend however to minimize grazing in order to allow greater control of the diet and optimize grassland utilization (van den Pol-van Dasselaar et al., 2008). Hay represents a common alternative to fresh grass as it can be stored for prolonged periods of time, be easily integrated in total mixed rations (TMR) and does not transmit flavor to the milk (Shipe et al., 1962). Nonetheless, the decision “to graze or not to graze” is often arbitrary based on the farm size and management, breeding program and feed price, without taking into consideration the impact of the feeding strategy on rumen function, rumen microbiota, nutrient utilization, methanogenesis and ultimately on the environment (van den Pol-van Dasselaar et al., 2008). Differences between fresh grass and grass hay in terms of rumen digestion of nutrients have been studied using cannulated animals (Petit and Tremblay, 1992; Holden et al., 1994), and several books have been published describing nutritional aspects of forage utilization by ruminants (Minson, 1990; Givens et al., 2000). However, there are no reports to our knowledge that have linked rumen function and the rumen microbiome comparing grass vs. conserved forages.

Similarly, there is an increasing tendency to use antioxidants in ruminant feeds (Chikunya et al., 2004). Vitamin E (α-tocopherol) is a naturally-occurring antioxidant which is vital for the body's defense against free radicals in cell membranes and for an optimum immune function (McDowell, 1989). Unlike group B vitamins and vitamin K which can be synthesized by rumen micro-organisms, vitamin E needs to be supplied in the diet. Fresh grass, in comparison to grass hay, is naturally rich in vitamin E and should meet the recommended animal requirements (NRC, 2001). However, there is an increasing body of evidence indicating that supra-nutritional dietary levels of vitamin E improve the quality of the ruminant products. In particular, vitamin E supplementation minimizes lipid oxidation in the milk and meat reducing undesirable flavors in milk (MacPherson, 1994) and improving color stability and shelf life in red meat (Arnold et al., 1992). Studies using rumen batch cultures have suggested that vitamin E has a positive effect on rumen fermentation pattern and protozoal numbers (Naziroglu et al., 2002) although it seems that these effects are highly dependent on the form and dose of the vitamin E used and the type of diet considered (Tagliapietra et al., 2013).

Here it was hypothesized that vitamin E supplementation may have a greater impact on the rumen microbiota and fermentation in preserved vs. fresh forage due to differences in vitamin E content and nutrient availability. Therefore, this paper aims to investigate grass (GRA) and grass hay (HAY) utilization by the rumen microbes when fed alone or supplemented with vitamin E. A dose-response experiment was conducted to determine the most effective form and concentration of vitamin E (Experiment 1) while a multi-omics approach was adopted to understand the mode of action of these feeding strategies in a longer term rumen simulation technique (Rusitec) (Experiment 2). This approach aimed to link rumen function and the microbiome based on a comprehensive description of rumen fermentation, methanogenesis and microbial protein synthesis, as well as a detailed characterization of the bacterial and methanogen communities using Next Generation Sequencing.

## Materials and methods

### Experiment 1: *in vitro* batch incubations

All animal procedures were carried out according to the Home Office Scientific Procedures Act 1986 (PLL 40/3653; PIL 40/9798) and protocols were approved by the Aberystwyth University Ethical Committee. A dose-response experiment was conducted to identify the effects of two different vitamin E forms on gas production and fermentation pattern. DL-α-tocopherol (Sigma-Aldrich T3251) and commercial DL-α-tocopheryl acetate with 50% silica adsorbate were used (Frank Wright Trouw Nutrition, Ashbourne, UK). The experimental design was: 2 vitamin E forms × 4 doses (0.5, 5, 50, and 500 IU/L) × 4 inoculum replicates plus 4 controls (0 IU/L) and 4 blanks (rumen fluid without feed), making 40 bottles in total. Inoculum replicates where prepared from rumen fluid taken from 4 rumen-cannulated Holsten-Friesian cows fed at maintenance level. Cows were fed 80% perennial ryegrass hay and 20% concentrate. Rumen liquids were sampled before morning feeding, filtered through a double layer of muslin, diluted 2:1 with incubation solution (Theodorou et al., 1994) and anaerobically dispensed to 120 mL Wheaton bottles (50 mL per bottle) containing 400 mg DM of grass hay and 100 mg DM of commercial concentrate (**Table 2**). Diets were ground using a hammer mill with 1 mm^2^ sieve pore size prior to use.

Bottles were sealed and held in an incubator at 39°C getting a gentle mix before each sampling time. After 24 h incubation, fermentation parameters such as pH, ammonia, VFA and methane emissions were measured: after gas pressure excess was released a gas sample (0.5 mL) was taken for measuring methane concentration. A sample representing 5% of the bottle liquid content was taken by aspiration and divided in two: the first subsample (1.6 mL) was diluted with 0.4 mL deproteinizing solution (200 mL/L orthophosphoric acid containing 10 mM of 2-ethylbutyric acid as an internal standard) for VFA determination. The second subsample (0.8 mL) was diluted with 0.48 mL of trichloroacetate (25 g/L) for ammonia analysis. Gas production was measured at 2, 4, 6, 9, 12, 24, 48, 72, and 96 h using a semi-automated pressure transducer (Bailey & Mackey Ltd. Birmingham, UK).

Fermentable OM (FOM) was stoichiometricly calculated (Groot et al., 1998). For gas production (GP), pressure measurements were corrected for the background GP from blank bottles and converted to units of volume (mL) using the ideal gas law. Cumulative GP data were fitted to the predictive equation described by France et al. (2000):

$$\begin{array}{l} Y=A\left(\text{1}-e^{-ct}\right) \end{array}$$

where *Y* (mL) is the cumulative GP at time *t* (h), *A* is the asymptotic or potential GP (mL) and *c* is the GP rate (μL h^−1^).

In order to determine the most effective dose for each vitamin E form, data were analyzed according to the following model:

$$\begin{array}{l} Y_{ijk}=\mu +T_{i}+D_{j}+TD_{ij}+A_{k}+e_{ijk} \end{array}$$

where *Y*_*ijk*_ is the dependent, continuous variable, μ is the overall population mean, *T*_*i*_ is the fixed effect of the type of vitamin E (*T*_*i*_ = tocopherol vs. tocopheryl-acetate), *D*_*j*_ is the fixed effect of the dose (*D*_*j*_ = 0, 0.5, 5, 50, 500 g/L), *TD*_*ij*_ is their interaction, *A*_*l*_ is the random effect of the animal inoculum (*j* = 1, 2, 3, 4) and *e*_*ijkl*_ is the residual error. When significant effects were detected across the different doses, means were compared by Fisher's protected LSD-test (Genstat 15th Edition, VSN International, UK). Significant effects were declared at *P* < 0.05 and tendency to differences at *P* < 0.1.

### Measurement of protozoal activity *in vitro*

The effect of the same doses and forms of vitamin E on protozoal activity was measured based on the breakdown of ^14^C-labeled bacteria by rumen protozoa (Belanche et al., 2015a). To prepare labeled bacteria, a pure culture of *Streptococcus bovis* ES1 was incubated at 39°C for 24 h in medium number two (Hobson, 1969) containing ^14^C-leucine (7 kBq m/L in 8 mL tube). Labeled bacteria were harvested from the culture by centrifugation (3000 × g for 15 min) and washed twice with simplex type salt solution (Williams and Coleman, 1992) containing ^12^C-leucine (5 mM). Incubation was conducted in quadruplicate using rumen fluid from the same 4 cannulated cows. Rumen fluids were filtered, diluted in simplex type salt solution (1:1) and distributed anaerobically in Hungate tubes (7.5 mL) containing ^14^C-labeled bacteria (0.5 mL) and Vitamin E at 0, 0.5, 5, 50, and 500 IU/L. Incubation tubes were held stable in a water bath at 39°C with manual mixing every 20 min. Tubes were sampled at 0, 1, 2, 3, and 4 h; samples (0.5 mL) were acidified with 0.125 mL of trichloroacetic acid (250 g/L) and centrifuged (13,000 × g for 5 min). Supernatants (200 μL) were diluted with 2 mL of scintillation fluid (Optiphase Hisafe 2, Perkin Elmer, USA) and the amount of radioactivity released was determined by liquid-scintillation spectrometry (Hidex 300 SL, Lablogic Systems Ltd. Broomhill, UK). A simple linear regression was conducted for each tube to model the relationship between the percentages of radioactivity released (with respect to the ^14^C-bacterial inoculum) and the time (from 0 to 4 h). The slope of this trend-line indicated the bacterial degradation rate (as %/h) by the rumen protozoa and ultimately their activity. Data was analyzed as described in Experiment 1.

### Experiment 2: rumen simulation technique

The Rusitec incubation procedure was used (Czerkawski and Breckenridge, 1977). The experimental design consisted of 2 × 2 factorial arrangement of treatments with two types of forage [grass (**GRA**) vs. grass hay (**HAY**)] and 2 levels of vitamin E supplementation [non-supplemented (−) vs. supplemented (+)] giving 4 treatments (GRA−, GRA+, HAY−, HAY+). The form and inclusion rate of vitamin E used in this experiment was chosen based on the most effective dose observed in Experiment 1. Thus, the same synthetic DL-α-tocopheryl acetate with silica adsorbate that was used in Experiment 1 was mixed with the concentrate and dosed at 50 IU/d in (+) vessels. The experimental diets had a 80:20 forage:concentrate ratio (**Table 2**). Forage was obtained from a 3-year old ryegrass monoculture (*Lolium perenne* L. cv. AberMagic, Germinal, Lincoln, UK) sown in a silt/clay loam soil at Trawsgoed (52°34′N, 3°95′W). Plant material from the third harvest performed on the 3rd September 2014 was used with a target maturity of reproductive stage R1-index 3.1 which shows a visible spikelet of inflorescence emergence (Moore and Moser, 1995). Grass was cut at 14:00 h to 5 cm above soil level; 20 kg of fresh grass was immediately frozen and kept at −20°C until the experiment for GRA treatment, while another 20 kg was left to dry in the field for 48 h and finished in an air force oven at 25°C for 5 days to generate HAY treatment. Both forages were chopped to 2–4 cm lengths by passing through a garden shredder (Bosch AXT Rapid 2200, UK).

Experiment 2 consisted of a single incubation period including 16 vessels as experimental units. Thus, each treatment had 4 replicates which were randomly allocated to the vessels and inoculated with rumen liquid from 4 cows (as used in Experiment 1). Vessels had 800 mL of effective volume and were kept at 39°C with constant vertical agitation. On day 1 vessels were inoculated with rumen fluid diluted 1:1 with artificial saliva (McDougall, 1948), then artificial saliva was continuously infused at a dilution rate of 3.35%/h (equivalent to 645 mL/d) using a multichannel peristaltic pump (Watson-Marlow 200 series, Cornwall, UK). Squeezed rumen solids (60 g FM) were placed in nylon bags (110 × 60 mm, pore size 100 μm^2^) and incubated in each vessel for 1 day to provide solid-associated bacteria, while experimental feed was supplied in a second bag (11.25 g DM/d). On day 2, the bag with rumen solids was removed and substituted by a new feed bag, therefore two feed bags were present in each vessel at any time. For subsequent days, the bag that had remained 2 days in each vessel was squeezed and washed with 50 mL of artificial saliva. The washing liquid was returned to the vessel, and a new feed bag was inserted daily.

### Experimental procedure and sampling

The incubation trial consisted of 18 days, using the first 10 days for adaptation and the last 8 for sampling. Dry matter degradation, methane emissions and outflow of fermentation products was measured on days 10, 11, and 12. After 48 h incubation, nylon bags were removed, rinsed in cold water for 5 min and DM disappearance was calculated from the weight loss. Fermentation gases were collected in gas-tight bags (TECOBAG 5L, PETP/AL/PE-12/12/75, Tesseraux container GmbH, Germany) to measure total gas and methane production. Daily production of ammonia and VFA were measured in the overflow flasks with 10 mL of saturated HgCl_2_ (diluted 1:5) added to stop the fermentation.

Microbial protein synthesis was determined using ^15^N as a microbial marker (Carro and Miller, 1999). On day 10 vessels were infused with 3 mg/vessel of ^15^N, as (^15^NH_4_)_2_SO_4_ to label the ammonia-N pool. To label the microbial protein, from day 10 forwards ^15^N was added into the artificial saliva (3.7 mg/L). On days 13 and 14 residues from the feed bags were mixed with their associated effluents and homogenized in a blender for 1 min at low speed to reconstitute the total digesta. This digesta was divided in two: one portion (100 g) was frozen to measure the non-ammonia N (NAN) and vitamin E outflows, while the other portion (200 g) was used to isolate the total bacteria and the ammonia-N fractions (Belanche et al., 2016a).

To describe the fermentation pattern, on days 15, 16, and 17 vessel pH and redox potential were measured at 4 and 24 h after feeding. Fluid from within the vessels was sampled (15 mL) by aspiration and this sample was split into 4 subsamples: The 1st subsample (10 mL) was snap frozen in liquid N for microbial characterization. The rest of the samples were used for VFA (1.6 mL), ammonia (0.4 mL) and lactate (0.8 mL) determination as described in Experiment 1. For protozoal optical counting and classification only 24 h samples were considered (Dehority, 1993). The redox potential (E_*h*_) is the potential difference between a platinum electrode and a standard hydrogen electrode. Since the latter was replaced by an Ag-AgCl reference electrode (F-995 Redox FermProbe, Broadley-James Ltd, Bedford, UK), all records were corrected using the formula (Nordstrom, 1977):

$$\begin{array}{l} E_{h}=E_{0}+C \end{array}$$

where *E*_0_ (mV) is the potential of the platinum electrode and *C* is the potential of the Ag-AgCl reference electrode relative to the standard hydrogen electrode, (i.e., +198 mV at 39°C).

### Sample analyses

For feed analysis, dry matter (DM) content was determined by drying in an oven at 105°C for 24 h. Organic matter (OM) concentration was determined by heating at 550°C for 6 h in a muffle furnace. Nitrogen and carbon concentration was measured by the Dumas combustion method (Elementar analyser, Vario MAX cube, Hanau, Germany). Neutral-detergent (NDF) and acid-detergent fiber (ADF) were determined using the Automated Fiber Analyzer (ANKOM 2000, Macedon, USA). Methane emissions were determined by gas chromatography (ATI Unicam 610 Series, UK), ammonia concentration in the fermenters (Weatherburn, 1967) was measured using an automated spectrophotometer (ChemWell T, Astoria Pacific, Oregon, USA), VFA concentration was determined using Gas Chromatography (Richardson et al., 1989). Concentrations of D- and L-lactate were determined using the Enzytec™ D/L-Lactic Acid kit (R-Biopharm, Darmstadt, Germany). Vitamin E concentrations in feed and overflow samples were determined by HPLC after saponification and extraction into heptane (Jensen et al., 1999). Briefly, 0.5 g DM of finely ground samples were suspended in a mixture of 24 mL ethanol (96%), 9 mL methanol, 10 mL aqueous ascorbic acid (200 g/L) and 7 mL KOH (500 g/L). Samples were saponified for 30 min at 80°C in the dark. Sample (1 mL) was then mixed with 0.25 mL of water and 2.5 mL of heptane and centrifuged at 1200 × g for 10 min. The top phase (heptane) was transferred to a new tube and 2.5 mL of heptane was added to the remaining phase. Centrifugation was repeated and both heptane fractions were pooled. The column used for vitamin E determination was a 4.6 × 150 mm Ace 5 sil column (Advanced Chromatography Technologies, Aberdeen, UK) fitted into an HPLC system (Agilent 1100 series, Waldbronn, Germany). Heptane modified with propanol (3 mL/L) constituted the mobile phase (1.5 mL/min) and florescence detection was set to wavelengths of 294 and 327 nm for excitation and emission, respectively. Identification and quantification of the vitamin E was determined by comparison of retention times and the peak areas using external standards (α-tocopherol, Sigma-Aldrich T3251).

### DNA extraction and quantitative PCR (qPCR)

Genomic DNA was extracted from vessel samples withdrawn at 4 and 24 h after feeding over a 3 day period. Samples were pooled by time point and lyophilized. Dry samples (100 mg DM) were bead-beaten for 1 min and DNA was extracted with a QIAamp DNA Stool Mini Kit (Qiagen Ltd, Crawley, UK) following the manufacturer's instructions, but with the temperature increased to 95°C for 10 min to maximize microbial lysis in the initial incubation. Dilution factors during the DNA extraction were considered in order to calculate the concentration of each microbial group per unit of initial sample. Genomic DNA quality and concentration were determined using the Nanodrop ND-100 spectrophotometer (Thermo-Scientific, USA).

Absolute concentration of rDNA copies from total bacteria, anaerobic fungi and methanogens were determined by qPCR and serial dilutions of their respective standards (10^−1^ to 10^−5^) as previously described (Belanche et al., 2016b). Briefly, qPCR was conducted in triplicate using a LightCycler® 480 System (Roche, Mannheim, Germany). Samples were prepared in 384-well plates using the Epimotion 5075 Liquid Handling System (Ependorf®, Stevenage, UK). Amplification reaction (12.5 μL) contained DNA template (1 μL), 1 mM of each primer and 6.25 μl of SYBR Green JumpStart Taq ReadyMix (Sigma-Aldrich Ltd, Dorset, UK). Amplification conditions were 95°C for 5 min, then 60 cycles at annealing temperatures described in Supplementary Table 1 for 30 s, 72°C for 30 s and 95°C for 15 s, and a final melting analysis was performed to determine primer specificity.

### Ion torrent next generation sequencing (NGS)

Rumen bacteria and methanogenic archaea communities were studied using NGS (de la Fuente et al., 2014). For bacterial sequencing, amplification of the V1-V2 hypervariable regions of the 16S rRNA was performed using bacterial primers (27F and 357R) followed by adaptors (Supplementary Table 2). For methanogen sequencing, amplification of the V2-V3 hypervariable region of the 16S rRNA was carried out using archaeal primers (86F and 519R) and adaptors (Supplementary Table 1). Forward primers were barcoded (10 nucleotides) for sample identification. PCRs had a total volume of 25 μl containing DNA template (1 μl), primers (0.2 μM of each) and 12.5 μL of KAPA HiFi Mix (Kapa Biosystems Ltd., London, UK). Amplification conditions for bacteria and methanogens were 95°C for 3 min, then 25 cycles (35 for methanogens) of 98°C for 20 s, 65°C for 20 s and 72°C for 30 s with a final extension step of 72°C for 5 min. Amplicon quality was assessed on a 1% agarose gel and purified using Agencout AMpure XP beads (Beckman Coulter Inc., Fullerton, USA). The DNA concentrations were determined in an Epoch Microplate Spectrophotometer (BioTek, Potton, UK) to pool equal amounts of each sample. Libraries were purified using the E-Gel System with 2% agarose gel (Life Technologies Ltd, Paisley, UK). Library quality and concentration was determined on an Agilent 21000 Bioanalyzer with a High Sensitivity DNA chip (Agilent Technologies Ltd., Stockport, UK). The emulsion PCRs were conducted using the Ion PGM Template OT2 400 Kit, then sequencing was carried out in an Ion Torrent Personal Genome Machine (PGM) system using Ion PGM Sequencing 316™ v2 and 314™ v2 chips for bacteria and methanogens, respectively (Life Technologies Ltd, Paisley, UK).

Following sequencing, data were processed as previously described (Belanche et al., 2016b). Briefly, sample identification numbers were assigned to multiplexed reads using the MOTHUR software environment. Data were de-noised by removing low quality sequences, sequencing errors and chimeras (quality parameters: maximum 10 homo-polymers, Q15 average over a 30 bp window, no mismatches allowed with the barcode and 1 maximum with the primer; Chimera check, both de-novo and database driven using Uchime). Due to the presence of a high proportion of short bacterial sequences with low quality in the terminal fraction (most of which clustered into the same OTU) bacterial reads were trimmed at 320 bp length (380 bp for methanogens). Sequences were then clustered into OTUs at 97% identity using CD-HIT-OTU (http://weizhong-lab.ucsd.edu/cd-hit-otu/). The number of reads per sample was normalized to the sample with the lowest number of reads. Ribosomal Database Project-II was used to obtain the bacterial taxonomic information based on 16S data, while RIM-DB database was used for methanogens (Seedorf et al., 2014). To exclude potential bacterial sequences from the methanogen dataset, methanogen sequences were also blasted with the Ribosomal Database Project-II and those which matched were further removed. Only annotations with a bootstrap value over 50% were assigned, otherwise they were considered as unclassified. Raw sequence reads were deposited at the EBI Short Read Archive from the European Nucleotide Archive (accession number PRJEB12948).

### Calculations and statistical analyses

Organic matter fermentation of total hexoses (FOM) as well as metabolic hydrogen production, hydrogen incorporation into fermentation products and hydrogen recovery were calculated from the stoichiometry of reducing equivalents released in acetate (*Ac*), propionate (*Pr*) butyrate (*But*), valerate (*Val*) and methane (*CH*_4_) (Marty and Demeyer, 1973) as:

$$\begin{array}{l} FOM=0.5Ac+0.5Pr+But+Val\\ \left[H\right]\;produced=2Ac+Pr+4But+3Val\\ \left[H\right]\;incorporated=2Pr+2But+4Val+CH_{4}\\ \left[H\right]\;recovery=100\times \left[H\right]incorporated:\left[H\right]produced \end{array}$$

Microbial N contribution to overflow and the ammonia incorporation by the bacteria were determined based on their ^15^N enrichment ratios, as follow:

$$\begin{array}{lll} Microbial\;N:NAN & = & Digesta\;NAN^{15}N\;enrichment:\\ Total\;bacteria^{15}N\;enrichment\\ Microbial-N\;from\;NH_{3} & = & Total\;bacteria^{15}N\;enrichment:\\ NH_{3}^{15}N\;enrichment \end{array}$$

Quantitative PCR data were log-transformed to assume normality. For rumen fermentation products, qPCR and microbial diversity, data were analyzed using an ANOVA as follows:

$$\begin{array}{l} Y_{ijk}=\mu +F_{i}+V_{j}+FV_{ij}+T_{k}+A_{l}+e_{ijkl} \end{array}$$

where *Y*_*ijk*_ is the dependent, continuous variable (*n* = 4), μ is the overall mean, *F*_*i*_ is the fixed effect of the forage (*i* = GRA vs. HAY), *V*_*j*_ is the fixed effect of the vitamin E supplementation (*j* = − vs. +), *FV*_*ij*_ is their interaction, *T*_*k*_ is the random effect of the sampling time (*k* = 2 h vs. 24 h), *A*_*l*_ is the random effect of the animal inoculum (*j* = 1, 2, 3, 4) and *e*_*ijkl*_ is the residual error. For feed disappearance and microbial protein synthesis data, the time effect was not included in the statistical analysis. Comparison among means was conducted as described in Experiment 1.

Treatment effects on NGS log-transformed data were determined based on their Bray-Curtis distance metric using the UPGMA function. Data were then analyzed by non-parametric permutational multivariate analysis of variance using PRIMER-6 software (PRIMER-E Ltd., Plymouth, UK). Statistical signification was calculated after 999 random permutations of residuals under a reduced model using the Monte Carlo test. A canonical correspondence analysis (CCA) was also conducted to explore the relationships between the structure of the bacterial and methanogen communities and the fermentation pattern. The signification of each variable was also calculated using 999 random permutations (R statistics; Vegan package). Bacterial and methanogen biodiversity indexes were calculated using normalized data. For bacterial and methanogen relative abundances data were tested for normality and homogeneity, then data were log transformed and *P*-values were adjusted for multiple testing to decrease the False Discovery Rate (Benjamini and Hochberg, 1995).

## Results

### Experiment 1: *in vitro* batch incubations

Inclusion of different forms of supplementary vitamin E in batch cultures at levels up to 500 IU/L had moderate effects on the fermentation pattern (Table 1). Values for pH, ammonia and methane emissions were unaffected by the experimental treatments, however inclusion of vitamin E increased total VFA concentrations (*P* < 0.001), FOM (*P* < 0.001), asymptotic GP (*P* = 0.037) and GP rate (*P* = 0.011), while decreasing the methane emissions per unit of FOM (*P* = 0.012) in respect to the control. The α-tocopherol promoted a greater increase in the asymptotic GP (*P* = 0.035) than α-tocopheryl acetate, however this later form of vitamin E incubated at concentrations above 50 IU/L tended to shift VFA production increasing the proportion of acetate (T × D, *P* = 0.040) in detriment to butyrate (T × D, *P* = 0.090). Inclusion of 50 IU/L promoted the greatest increase in total VFA (+33%) and FOM (+32%) and the lowest methane per unit of FOM (−24%). In terms of protozoal activity, the amount of bacteria degraded by protozoa increased linearly during the incubation. Inclusion of α-tocopheryl acetate increased the protozoal activity in comparison with α-tocopherol (*P* = 0.015), with the effect dose-independent.

**Table 1:** **Effect of different forms and doses of vitamin E on the rumen function and protozoal activity in rumen batch cultures (Experiment 1)**.

**Dose (IU/L)**	**α-tocopherol**					**α-tocopheryl acetate**					**SED^3^**	***P*****-value**		
	**0**	**0.5**	**5**	**50**	**500**	**0**	**0.5**	**5**	**50**	**500**		**Type**	**Dose**	**T × D**
pH	6.36	6.34	6.36	6.36	6.34	6.36	6.37	6.35	6.35	6.34	0.023	0.776	0.644	0.793
NH_3_-N (mg/dL)	19.6	21.0	22.2	20.2	21.3	19.6	22.1	19.9	20.6	21.4	2.440	0.899	0.793	0.893
Total VFA (mM)	62.0	73.4	78.1	72.6	68.5	62.0	69.7	73.3	80.6	75.6	4.510	0.516	<0.001	0.163
Acetate (%)	67.7^bc^	68.2^ab^	68.1^abc^	67.9^abc^	67.6^bc^	67.7^bc^	67.8^abc^	67.3^c^	68.5^a^	68.5^a^	0.394	0.748	0.253	0.040
Propionate (%)	16.4	16.5	16.6	16.6	16.7	16.4	16.5	16.7	16.5	16.4	0.140	0.551	0.079	0.428
Butyrate (%)	11.6	11.3	11.1	11.3	11.5	11.6	11.5	11.5	11.0	11.1	0.217	0.982	0.116	0.090
Asymptotic GP (mL)	116	119	120	120	124	116	117	120	117	119	2.155	0.035	0.037	0.441
GP rate (μL/h)	64.5	64.8	67.3	69.0	68.3	64.5	65.6	67.0	64.5	67.5	1.400	0.144	0.011	0.102
FOM^1^ (mg)	309	366	388	361	342	309	348	365	401	376	22.27	0.513	<0.001	0.178
Methane (mmol/d)	0.90	0.91	0.94	0.93	0.94	0.90	0.91	0.94	0.89	0.93	0.025	0.337	0.239	0.786
Methane (mmol/g FOM)	2.99	2.51	2.42	2.60	2.76	2.99	2.64	2.57	2.23	2.49	0.224	0.488	0.012	0.384
Protozoal activity^2^ (%/h)	9.95	9.58	9.36	9.46	9.53	9.95	10.26	10.41	9.95	9.66	0.404	0.015	0.677	0.376

1 Fermentable OM stoichiometrically calculated based on VFA production (Marty and Demeyer, 1973).

2 Protozoal activity determined in vitro as the percentage of ^14^C-labeled bacteria degraded by rumen protozoa.

3 Standard error of the difference among means for the interaction T × D (n = 4). Within a row means without a common superscript differ (P < 0.05).

### Experiment 2. rumen simulation technique: feed degradability and fermentation pattern

Grass had a slightly higher N content than grass hay, while the opposite was true in terms of NDF and ADF concentrations (Table 2). As expected, grass had a much higher vitamin E concentration (2.35-fold) than grass hay. In terms of diet degradability (Table 3), HAY diets tended to promote a greater disappearance of NDF (*P* = 0.076) and ADF (*P* = 0.016). HAY diets also had a greater gas (*P* = 0.009) and methane production (*P* = 0.012) than GRA diets, but similar levels of VFA production. Stoichiometry calculations showed that HAY, in comparison to GRA, had slightly greater metabolic H production (*P* = 0.093) but much greater H incorporation (*P* = 0.008) and recovery (*P* = 0.018), as well as greater CH_4_:VFA ratio (*P* = 0.033) (Table 3). Vitamin E supplementation also tended to increase the disappearance of OM, C and NDF (*P* < 0.09). Moreover vitamin E supplementation tended to decrease metabolic H recovery and methane emissions per g of degradable OM and per unit VFA production in GRA but not in HAY diets (interaction F × V, *P* < 0.1, Table 3).

**Table 2:** **Chemical composition of the experimental diets (in % of DM unless stated)**.

	**Grass**	**Grass hay**	**Concentrate^a^**
Dry matter (% FM)	17.5	84.9	88.6
Organic matter	91.2	91.0	93.8
Nitrogen	1.83	1.69	2.51
Carbon	43.8	43.5	44.7
Neutral detergent fiber	50.0	54.6	39.3
Acid detergent fiber	24.0	28.1	12.1
Vitamin E (IU/kg DM)	72.3	30.8	ND

a Commercial concentrate made of: wheat 44.25, barley 15, palm kernel expeller 14, rapeseed expeller 11.7, maize meal 7.5, wheat-feed 5, limestone flour 1.95, NaHCO_3_ 0.3, NaCl 0.15 and NH_4_Cl 0.15% in DM. ND, not detected (Celtic Pride Premium Beef Nuts, UK).

**Table 3:** **Effect of the type of forage and vitamin E supplementation on feed degradability and methanogenesis in the Rusitec system**.

**Forage**	**Grass**		**Grass hay**		**SED^a^**	***P*****-value**		
**Vitamin E**	**GRA−**	**GRA+**	**HAY-**	**HAY+**		**F**	**V**	**F × V**
**DISAPPEARANCE (%)**								
OM	57.4	63.5	60.6	63.7	3.34	0.483	0.084	0.532
C	58.3	63.8	61.9	64.5	3.02	0.340	0.090	0.507
NDF	44.1	52.5	52.8	55.1	3.99	0.076	0.089	0.307
ADF	33.0	40.0	44.8	47.6	4.61	0.016	0.170	0.541
**GAS EMISSIONS**								
Total gas (L/d)	1.72	1.71	1.82	2.03	0.092	0.009	0.167	0.133
Methane (mM)	2.97	2.92	3.30	3.73	0.310	0.030	0.416	0.308
Methane (mmol/d)	5.10	5.01	6.04	7.59	0.792	0.012	0.223	0.180
Methane (mmol/g Deg.OM)	0.87	0.77	0.96	1.15	0.105	0.011	0.584	0.091
[H] produced^b^ (mmol/d)	58.2	64.0	67.8	67.9	5.100	0.093	0.432	0.450
[H] incorporated^b^ (mmol/d)	50.5	52.9	60.8	66.0	4.840	0.008	0.298	0.685
[H] recovery^b^ (%)	87.3	82.7	89.6	97.4	4.160	0.018	0.588	0.064
CH_4_:VFA (mol/mol)	0.165	0.149	0.172	0.214	0.020	0.033	0.405	0.069

a Standard error of the difference for the interaction between the type of forage and the vitamin E supplementation at 50 IU/d (F × V, n = 4).

b Metabolic hydrogen stoichiometrically calculated based on VFA production (Marty and Demeyer, 1973).

In terms of fermentation pattern, vessels fed GRA diets had a lower pH (*P* = 0.003) and greater concentrations of ammonia (*P* = 0.003), lactate (*P* < 0.001), D/L lactate ratio (*P* = 0.007) and redox potential (*P* = 0.003) than those fed HAY diets (Table 4). Total VFA concentration was constant across treatments, but HAY diets promoted a greater molar proportion of acetate (*P* = 0.042) and branched chain volatile fatty acids (iso-butyrate and iso-valerate) while GRA diets increased propionate molar proportion (*P* < 0.001). Vitamin E supplementation tended to decrease butyrate molar proportion (*P* = 0.076) and to promote greater branched-chain fatty acid values in HAY+ than in GRA+ diets (interaction F × V, *P* = 0.054), (Table 4). Quantitative PCR revealed a positive effect of vitamin E supplementation (*P* = 0.021) and GRA diets (*P* = 0.046) on the concentration of total bacteria (Table 4). However, no differences were observed in the abundance of anaerobic fungi and methanogens.

**Table 4:** **Effect of the type of forage and vitamin E supplementation on rumen fermentation pattern in the Rusitec system**.

**Forage**	**Grass**		**Grass hay**		**SED^a^**	***P*****-value**		
**Vitamin E**	**GRA−**	**GRA+**	**HAY−**	**HAY+**		**F**	**V**	**F × V**
**FERMENTATION PATTERN**								
pH	6.69	6.65	6.76	6.75	0.036	0.003	0.349	0.587
E*_*h*_* redox potential (mv)	−109	−106	−113	−116	2.980	0.003	0.807	0.144
Ammonia-N (mg/dL)	6.94	7.31	5.88	5.99	0.306	<0.001	0.273	0.550
Total VFA (mM)	43.4	43.8	43.4	43.3	2.229	0.877	0.934	0.851
**MOLAR PROPORTION (%)**								
Acetate	41.3	42.2	42.7	42.8	0.652	0.042	0.304	0.423
Propionate	21.6	21.9	24.7	24.5	0.922	<0.001	0.897	0.742
Butyrate	20.8	19.0	20.0	19.9	0.713	0.888	0.076	0.102
Branched-chain VFA	4.29	4.09	4.31	4.48	0.126	0.027	0.874	0.054
Lactate (mM)	4.53	5.69	2.01	2.66	0.915	<0.001	0.176	0.703
D/L ratio	0.86	0.81	0.70	0.45	0.127	0.007	0.109	0.288
**MICROBIAL NUMBERS (log copy/g DM)**								
Bacteria	8.48	8.61	8.30	8.50	0.096	0.046	0.021	0.660
Anaerobic fungi	2.92	2.91	2.84	2.99	0.172	0.990	0.573	0.500
Methanogens	1.36	1.35	1.17	1.49	0.134	0.766	0.110	0.089
Methanogens (10^3^ × ΔC_T_)	0.23	0.14	0.18	0.23	0.769	0.758	0.728	0.193

a Standard error of the difference for the interaction between the type of forage and the vitamin E supplementation at 50 IU/d (F × V). Samples were taken at 4 and 24 h after feeding (n = 8).

Protozoa counts were greater in vessels fed HAY than those fed GRA (*P* < 0.001), (**Table 6**). Moreover, HAY vessels tended to have a greater proportion of small protozoa (Subfamily *Entodiniinae, P* = 0.083) and a lower proportion of big protozoa (Subfamily *Diplodiniinae, P* = 0.003). Vitamin E supplementation tended to increase total protozoal numbers (*P* = 0.075) but had no effect on the abundance of the different protozoal groups.

### Bacterial 16S rDNA sequencing

**Figure 1:**
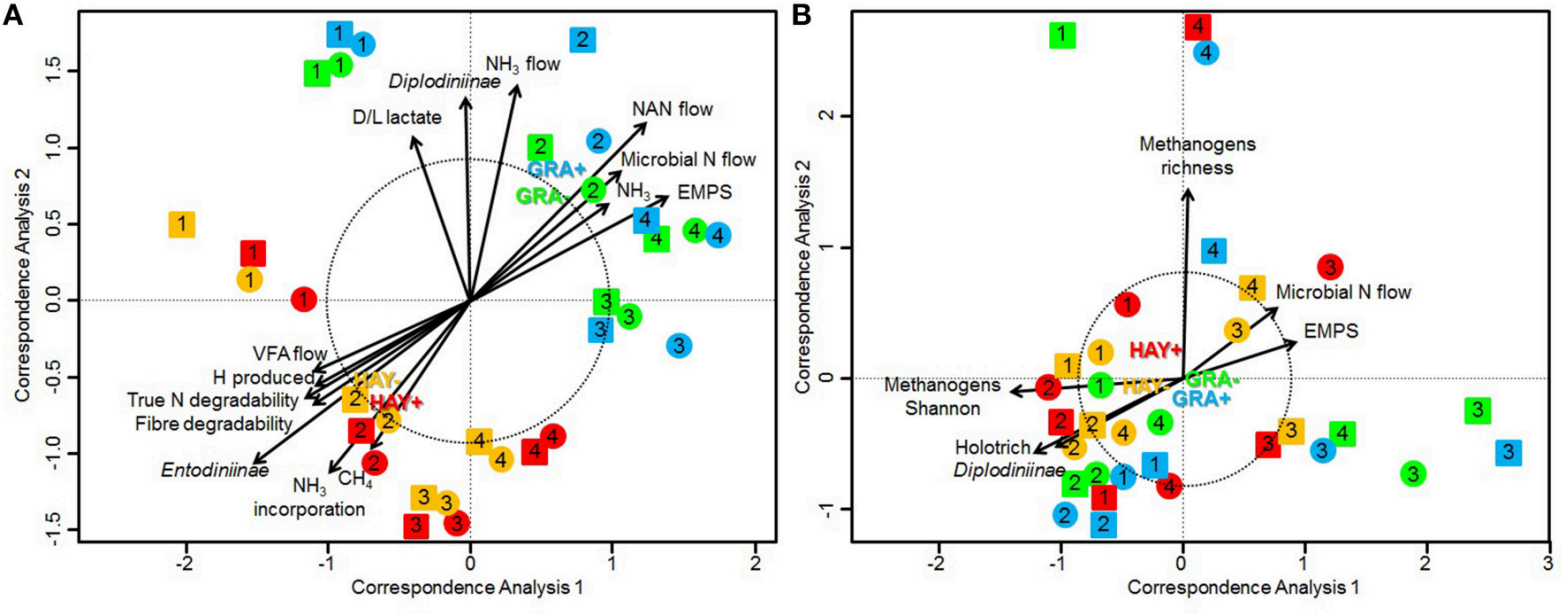
**Canonical correspondence analysis illustrating the effect of grass (GRA), grass hay (HAY) and vitamin E supplementation (−, +) on the relationship between the structure of the bacterial community (A) or methanogen community (B) with the rumen function in the Rusitec system**. Arrows show the direction of the gradient and their length is proportional to the correlation. Arrows longer that the dotted circle are significant (*P* < 0.05). Centroid is indicated for each treatment: GRA– (green), GRA+ (blue), HAY– (yellow), HAY+ (red). Circles and squares represent samples taken at 4 and 24 h after feeding, respectively.

Bacterial sequencing generated 4.33 million raw sequences. Quality filtering resulted in 659,378 high quality sequences (320 bp long) which clustered in 971 different OTUs with 10,572 reads per sample after normalization. Permutational analysis of variance (Table 5) revealed a strong effect of the type of forage on the structure of the bacterial community (*P* = 0.037), but no effect was observed with vitamin E supplementation nor for F × V interaction (Supplementary Figure 1). The effects of the animal used as inoculum (*P* = 0.001) and the time points (*P* = 0.010) also had a significant impact on the structure of the bacterial community. A CCA was performed in order to detect possible correlations between the structure of the bacterial community (samples) and the rumen fermentation parameters (Figure 1A). This analysis showed a clear separation of the GRA (top right) and HAY (bottom left) samples in the ordination plot. Several variables were correlated with the sample distribution: levels of *Diplodiniinae* protozoa (*P* = 0.002) and ammonia (*P* = 0.004), flows of ammonia-N (*P* < 0.001), NAN (*P* = 0.001) and microbial N (*P* = 0.001) as well as efficiency of microbial protein synthesis (EMPS, *P* = 0.001) were positively correlated to the structure of the bacterial community in GRA samples. On the contrary, concentration of *Entodiniinae* protozoa (*P* = 0.001), degradability of N (*P* < 0.001) and NDF (*P* < 0.001), production of VFA (*P* = 0.004), metabolic H (*P* = 0.002) and methane (*P* = 0.002) and ammonia incorporation by the bacteria (*P* = 0.001) were positively correlated with HAY samples (Figure 1A).

**Table 5:** **PERMANOVA illustrating the effect of the type of forage and vitamin E supplementation on the structure of the bacterial and methanogen communities in the Rusitec system**.

**Community^a^**	**Bacteria**		**Methanogens**	
	**Pseudo-F**	***P*-value**	**Pseudo-F**	***P*-value**
Forage	3.92	0.037	2.80	0.082
Vitamin E	1.38	0.283	2.83	0.103
Forage × Vitamin E	1.11	0.366	0.70	0.575

a Higher Pseudo-F and lower similarities and P-values correspond to greater differences in the microbial composition. Samples were taken at 4 and 24 h after feeding (n = 8).

In terms of bacterial diversity (Table 6), no differences were observed across diets for the Chao index or Good's coverage, indicating that the sequencing depth was comparable across treatments. HAY diets increased most of the bacterial diversity indices (i.e., richness, Shannon and Evenness) indicating the presence of a greater number of bacterial species and similar abundance of the different bacterial species (OTUs) compared to GRA diets. On the contrary, vitamin E supplementation decreased all diversity indices (*P* < 0.039).

**Table 6:** **Effect of the type of forage and vitamin E supplementation on rumen biodiversity indices of the bacterial, methanogen and protozoal communities in the Rusitec system**.

**Forage**	**Grass**		**Grass hay**		**SED^a^**	***P*****-value**		
**Vitamin E**	**GRA−**	**GRA+**	**HAY−**	**HAY+**		**F**	**V**	**F × V**
**BACTERIA**								
Richness	632	616	688	635	22.22	0.025	0.039	0.252
Shannon	4.68	4.53	5.07	4.60	0.119	0.011	0.001	0.073
Evenness	0.77	0.71	0.78	0.71	0.017	0.024	0.002	0.097
Simpson	0.97	0.96	0.98	0.96	0.008	0.113	0.021	0.213
Chao	848	802	879	905	80.50	0.250	0.859	0.533
Good's coverage	0.71	0.70	0.73	0.68	0.028	0.900	0.106	0.301
**METHANOGENS**								
Richness	13.3	13.1	14.0	14.4	0.536	0.014	0.745	0.516
Shannon	1.72	1.62	1.76	1.72	0.037	0.011	0.015	0.24
Evenness	0.67	0.63	0.67	0.65	0.014	0.334	0.012	0.44
Simpson	0.75	0.73	0.76	0.75	0.011	0.049	0.035	0.51
Chao	13.4	13.9	15.8	14.9	0.946	0.017	0.712	0.314
Good's	0.92	0.87	0.86	0.89	0.033	0.455	0.786	0.119
**PROTOZOA^b^**								
Total (log cells/mL)	3.75	3.90	4.06	4.08	0.137	<0.001	0.075	0.160
Subf. *Entodiniinae* (%)	65.4	64.6	73.8	73.4	6.250	0.083	0.989	0.963
Subf. *Diplodiniinae* (%)	1.98	2.34	0.30	0.65	0.604	0.003	0.427	0.997
*Isotricha* (%)	0.17	0.13	0.00	0.22	0.205	0.768	0.551	0.406
*Dasytricha* (%)	32.4	33.9	25.9	25.7	5.94	0.136	0.966	0.939

a Standard error of the difference for the interaction between the type of forage and the vitamin E supplementation at 50 IU/d (F × V). Samples were taken at 4 and 24 h after feeding (n = 8).

b Protozoal samples were taken before feeding and measured by optical microscopy (n = 4).

**Figure 2:**
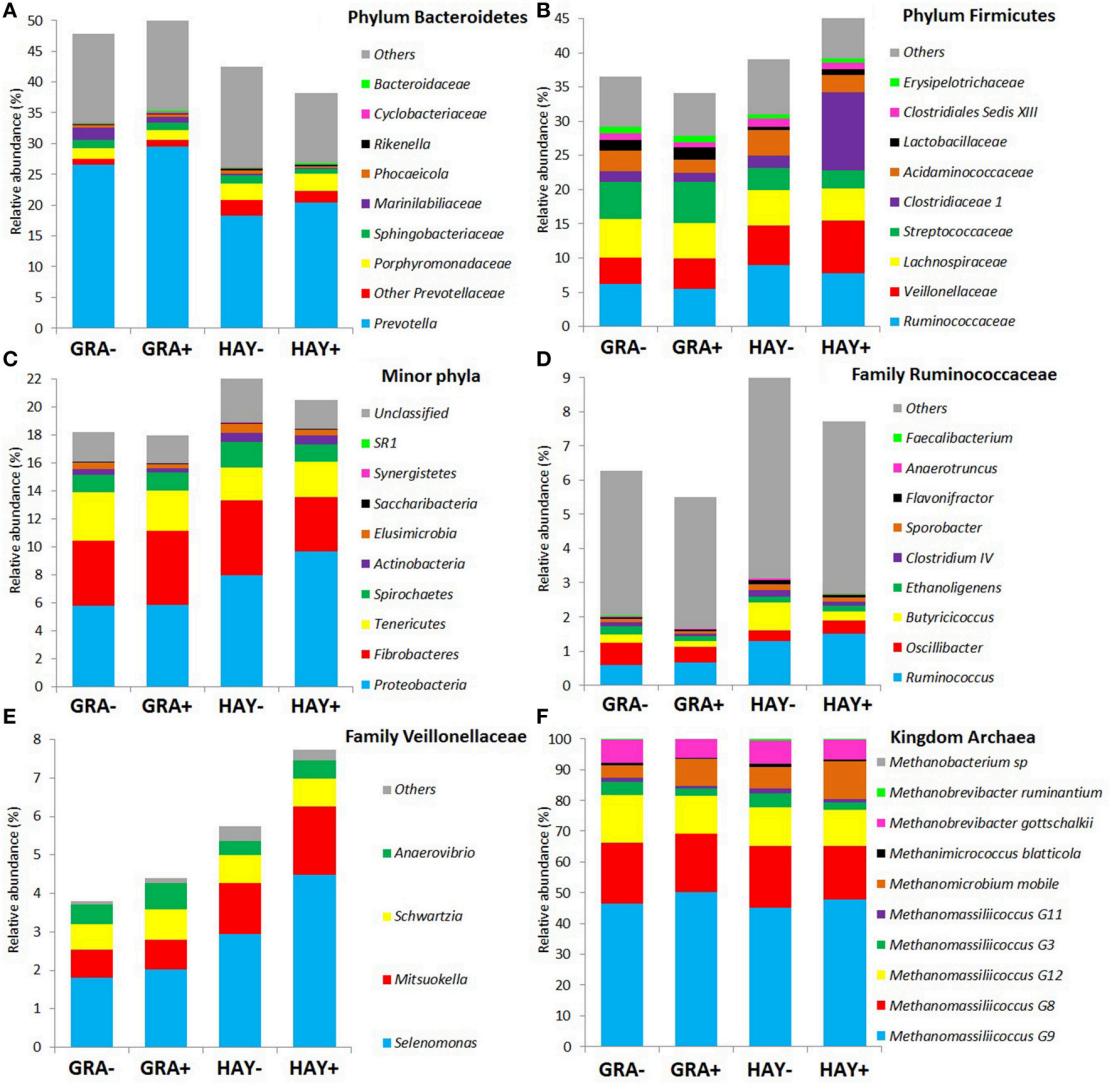
**Effect of the type of forage and vitamin E supplementation on the abundance of the main bacterial (A–E) and archaeal (F) phyla and families in the Rusitec system**. Samples were taken at 4 and 24h after feeding (*n* = 8).

Based on the classification by RDPII, Bacteroidetes was the most abundant phylum across diets (45%) followed by Firmicutes (39%), Fibrobacteres (4.8%), Proteobacteria (4.0%), Tenericutes (2.8%), Spirochaetes (1.4%), and minor phyla (1.1%) whereas few sequences were unclassified at the phylum level (2.4%), (Figure 2). The type of forage had an effect on the abundance of the main phyla (Figure 2 and Supplementary Table 2): GRA diets increased the abundance of Bacteroidetes, particularly the families *Prevotellaceae* and *Marinilabiliaceae*, as well as the phyla Tenericutes (family *Anaeroplasmataceae*). On the contrary HAY diets increased the abundance of Firmicutes and its main families (*Ruminicoccaceae, Veillonellaceae*, and *Clostridiales*) and other minor phyla such as Proteobacteria, Spirochaetes, Elusimicrobia and Acinobacteria (Figure 2). The effect of vitamin E supplementation on the abundance of the different bacterial groups was less obvious and only lowered the concentration of the phylum Elusimicrobia, the family *Marinilabiliaceae* (phylum Bacteroidetes) and the genus *Acidaminococcus* (phylum Firmicutes) in comparison to non-supplemented diets (Figure 2). No interactions F × V where observed for any of the bacterial groups studied.

### Methanogen 16S rDNA sequencing

Methanogen sequencing generated 0.75 million raw sequences. Quality filtering and removal of bacterial sequences resulted in 116,381 high quality methanogen sequences (average length of 380 bp) that were clustered in to 18 unique OTUs with 1765 sequences per sample after normalization. Permutational analysis of variance showed a tendency for the type of forage (*P* = 0.082) and vitamin E supplementation (*P* = 0.10) to influence the methanogen community (Table 5). The structure of this community was however mainly determined by the effect of the animal used as inoculum (*P* = 0.002). As a result CCA analysis showed no clear separation of the samples in the ordination plot according to the experimental treatments (Figure 1B). Moreover, the structure of the methanogen community was not correlated with most of the fermentation parameters. Only the methanogen diversity (richness and Shannon index, *P* = 0.001), the type of rumen protozoa (Holotrich *P* = 0.007; *Diplodiniinae P* = 0.024) and the efficiency of microbial protein synthesis (*P* = 0.024) were correlated with the structure of this community, with these correlations not being driven by the experimental diets (Figure 1B).

Similar to bacteria, methanogen 16S rDNA sequencing showed no differences in Good's coverage, indicating a homogeneous sequencing depth across treatments (Table 6). HAY diets had a greater methanogen diversity in comparison to GRA diets for most of the indices studied (*P* < 0.05) but not for evenness indicating a decrease in the number of methanogen species but maintaining similar abundance across species. On the contrary diet supplementation with vitamin E maintained the number of methanogen species (richness) but promoted an uneven abundance distribution across them (lower Shannon, Evenness and Simpson, *P* < 0.05).

Based on the RIM-DB database, only 10 species belonging to 4 families made up the entire methanogen population (Supplementary Figure 2): *Methanomassiliicoccaceae, Methanobacteriaceae, Methanomicrobiaceae*, and *Methanosarcinaceae*. The type of forage had a minor effect on the abundance of the main methanogen groups and only *Methanobacterium* (*P* = 0.016) and *Methanimicrococcus* levels (*P* = 0.002) were higher in vessels fed HAY instead of GRA diets. On the contrary, diet supplementation with vitamin E tended to decrease the abundance of *Methanomassiliicoccaceae* (*P* = 0.085), *Methanobacteriaceae* (*P* = 0.080) and *Methanosarcinaceae* (*P* = 0.020), while increased the abundance of *Methanomicrobiaceae* (*P* < 0.001) (Supplementary Table 3).

### Fermentation products and microbial protein synthesis

Similar daily yields of total VFA were observed across treatments (Table 7). HAY diets increased propionate (*P* = 0.047) outflow as well as apparent (*P* = 0.002) and true N digestibility (*P* = 0.015) and the ammonia uptake by the rumen bacteria (*P* < 0.001). GRA diets lead to increased overflows of ammonia (*P* = 0.003), NAN (*P* < 0.001), NANM-N (*P* = 0.007), microbial N (*P* = 0.003), as well as greater efficiency of microbial protein synthesis per unit of N intake (*P* = 0.032), degradable OM (*P* < 0.001) and truly degraded N in the rumen (*P* = 0.003). Vitamin E supplementation had no substantial effect on the outflow of fermentation products or microbial protein but decreased the efficiency of microbial protein synthesis (per g of degradable OM, *P* = 0.027). Diet GRA+ had the greatest NAN outflow with most of the increase due to increased NANM-N flow (interaction F × V). A significant interaction F × V indicated that vitamin E supplementation of HAY diets had a greater impact on the vitamin E outflow than observed in GRA diets (F × V, *P* = 0.018), (Table 7).

**Table 7:** **Effect of the type of forage and vitamin E supplementation on fermentation products and microbial protein synthesis in the Rusitec system**.

**Forage**	**Grass**		**Grass hay**		**SED^1^**	***P*****-value**		
**Vitamin E^1^**	**GRA−**	**GRA+**	**HAY−**	**HAY+**		**F**	**V**	**F × V**
**FERMENTATION PRODUCTS (mmol/d)**								
Total VFA	31.2	33.8	35.1	35.5	2.374	0.131	0.383	0.536
Acetate	14.2	15.7	15.7	16.8	1.247	0.182	0.164	0.794
Propionate	8.02	8.93	9.60	9.96	0.801	0.047	0.293	0.631
Butyrate	4.50	4.94	5.51	5.04	0.483	0.139	0.975	0.211
Isobutyrate	0.39	0.41	0.39	0.41	0.036	0.950	0.433	0.947
Valerate	1.28	1.27	1.60	1.42	0.067	<0.001	0.066	0.107
Isovalerate	1.07	1.28	1.13	1.22	0.086	0.958	0.034	0.356
Ammonia N (mg/d)	58.8	58.3	48.8	46.5	3.86	0.003	0.616	0.755
**OUTFLOW (mg/d)**								
DM (g/d)	15.0	15.3	14.4	14.2	0.50	0.046	0.955	0.559
Vitamin E	2.27^c^	35.3^b^	0.60^c^	51.7^a^	4.44	0.043	<0.001	0.018
NAN	140	152	122	115	6.92	<0.001	0.609	0.084
NANM-N^2^	39.9^b^	54.8^a^	36.9^b^	28.6^b^	5.97	0.007	0.456	0.022
Microbial-N	100.5	97.7	84.9	86.3	4.71	0.003	0.840	0.536
**N Metabolism (%)**								
Rumen apparent N degradability	36.5	31.0	41.8	45.1	3.15	0.002	0.630	0.079
Rumen true N degradability	82.0^a^	75.2^b^	82.4^a^	86.4^a^	2.73	0.015	0.491	0.021
Microbial-N from ammonia	36.0	36.3	45.7	44.8	2.47	<0.001	0.875	0.746
**EFFICIENCY OF SYNTHESIS (g/g)**								
Microbial-N: NAN	0.72^ab^	0.64^b^	0.70^ab^	0.75^a^	0.033	0.094	0.726	0.024
Microbial-N: N intake	0.45	0.44	0.41	0.41	0.022	0.032	0.862	0.548
Microbial-N: true N degradable	0.55	0.59	0.49	0.48	0.031	0.003	0.622	0.269
Microbial-N: Deg.OM (mg/g)	17.0	14.9	13.6	13.2	0.644	<0.001	0.027	0.104

1 Standard error of the difference for the interaction between the type of forage and the vitamin E supplementation at 50 IU/d (F × V, n = 4). Within a row means without a common superscript differ (P < 0.05).

2 NANM-N; non-ammonia non-microbial N calculated by subtracting microbial N from non-ammonia N flow.

## Discussion

### Effect of vitamin E on the fermentation pattern

Vitamin E is a natural antioxidant present as eight tocopherol isomers which differ in biological activity (Shin and Owens, 1990). In practice, the content of the natural form (α-tocopherol) in feeds is variable and not readily predictable, thus animal feeds are commonly supplemented with a synthetic form (α-tocopheryl acetate) at the rate of 10–30 IU/kg (Shin and Owens, 1990). Under our experimental conditions, HAY had substantially lower α-tocopherol content (−2.3-fold) than GRA, which is in line with the reported degradation of vitamin E (−2.6-fold) during the haymaking process (Givens et al., 2000).

Experiment 1 revealed that dietary supplementation with vitamin E, either as α-tocopherol or as α-tocopheryl acetate, had a positive impact on the rumen fermentation in terms of VFA and gas production. A greater bacterial predation by protozoa was however observed in batch cultures incubated with α-tocopheryl acetate compared with α-tocopherol (+5%), possibly due to the greater stability described for the acetate form (Ballet et al., 2000). The most effective dose was 50 IU/L of vitamin E as α-tocopheryl acetate, which led to the greatest increase in total VFA (+33%), FOM (+32%) and the lowest methane production per unit of FOM (−24%), as well as to a small shift from butyrate to acetate production. In line with previous reports (Hino et al., 1993; Tagliapietra et al., 2013) no further improvements in the fermentation pattern were observed with higher vitamin E dosage (500 IU/d), possibly because vitamin E accumulation can induce a prolonged oxidative stress in the rumen (Weiss et al., 1995). The total concentration of vitamin E in the rumen which could impair the rumen function cannot however be defined from these results, since it would include the vitamin E supplementation, but also the feed and rumen inoculum contributions.

Based on these results, α-tocopheryl at 50 IU/d was chosen to investigate its mode of action in Experiment 2. This nutritional strategy tended to increase feed disappearance in terms of OM (+8%), NDF (+11%), and ADF (+13%). This observation agrees with the batch culture data and highlights the ability of vitamin E to create the environmental conditions which favor a more efficient feed degradation by the microbes. This extra energy supply may explain the observed increased in milk fat yield (+15%) observed in cows supplemented with 12,000 IU/d of vitamin E (Pottier et al., 2006). Similarly, an increase in the ruminal levels of VFA and body mass gain was reported in lambs supplemented vitamin E (250 IU/Kg DM) and selenium (0.3 mg/Kg DM) (Naziroglu et al., 1997).

### Effect of vitamin E on rumen microbial communities

Although the rumen is considered as an anaerobic environment, dissolved oxygen is present at concentrations as high as 3 mmol/L (Stewart and Bryant, 1988). Cellular membranes are susceptible to oxidation because of the accumulation of free radicals in the rumen (Naziroglu et al., 2002). As facultative and obligate anaerobes, rumen bacteria, protozoa, methanogens and fungi show different abilities to grow in the presence of oxygen (Lloyd et al., 1989). This sensitivity to oxygen, together with their ability to grow *in vitro*, may explain the differences in the abundances of the main bacterial and methanogen groups observed between *in vitro* (Belanche et al., 2016b) and *in vivo* studies (St-Pierre and Wright, 2013). Here, supplementation with vitamin E increased the concentration of total bacteria but decreased bacterial diversity (−34 OTUs) regardless of the type of forage used. Using batch cultures, a positive effect of vitamin E supplementation has been observed on the fermentation of hay diets but not for corn diets suggesting that digestion of forage is mainly operated by obligate anaerobe bacteria, whereas digestion of concentrate is operated by facultative anaerobe bacteria (Tagliapietra et al., 2013). However, addition of vitamin E did not change the redox potential in the vessels (an indicator of the oxygen level), and thus it neither modified the structure of the bacterial community nor the abundance of most bacterial species.

Similarly, vitamin E supplementation tended to increase the concentration of total protozoa (+0.10 log) without modifying the relative abundance of the main groups. Previous work has indicated that vitamin E supplementation has a positive effect on rumen protozoal numbers *in vitro* (Naziroglu et al., 2002) and *in vivo* (Naziroglu et al., 1997) favoring the growth of *Diplodinium* relative to *Dasytricha* in lambs. This author also reported an increase in rumen ammonia concentration (Naziroglu et al., 2002) and suggested that bacterial predation by protozoa would be stimulated by vitamin E supplementation in batch cultures (Wilsdorf et al., 1984). However, our results disprove this hypothesis, since the specific measurement of the bacterial predatory activity using ^14^C-labeled bacteria revealed no effect of vitamin E on the protozoal activity in respect to the control.

Methanogenesis in archaea is a form of anaerobic respiration in which the terminal electron acceptor is not oxygen but carbon and oxygen inhibits the growth of methanogens (Sharp et al., 1998). This study revealed that vitamin E supplementation tended to increase methanogen numbers in HAY diets but not in GRA diets, possibly because GRA- diet had already a substantial concentration of vitamin E *per se*. Moreover, this effect was species-specific promoting an increase in the abundance of certain methanogen groups (*Methanomicrobium mobile* and *Methanomassiliicoccus* group 9) and a decrease in others (*Methanobrevibacter, Methanosarcinaceae*, and *Methanomassiliicoccus* group 3 and 11). *Methanosarcinales* are the only methanogens with cytochromes and therefore can grow on the broadest range of substrates and conditions but at a slower rate and methane yield than those without cytochromes (Thauer et al., 2008). Moreover, methanogens most often associated with protozoa are from the orders *Methanobacteriales* and *Methanomicrobiales* (Sharp et al., 1998). Therefore, the impact of vitamin E supplementation on the methanogen community could be a combination of a direct effect of the vitamin E as an antioxidant and an indirect effect mediated by rumen protozoa. A more detailed characterization of other bioactive compounds present in forages, such as carotenes, tannins, saponins or flavonoids could help to better understand the impact of the forage preservation method on the rumen microbiome.

### Effect of vitamin E on rumen N metabolism

Overall no effect of vitamin E supplementation on microbial protein synthesis was noted. It seems that the greater bacterial growth in vessels supplemented with vitamin E was counterbalanced by the greater bacterial predation by rumen protozoa (Belanche et al., 2012a). This observation is supported by the increased rumen overflow of branched chain fatty acids with vitamin E (+12%) as an indicator of rumen proteolysis (Firkins, 1996). A similar lack of effect of vitamin E on rumen microbial protein synthesis was reported in sheep fed dried grass diets containing different types of oil (Chikunya et al., 2004). Our study however revealed that the effect of vitamin E supplementation on the N metabolism was diet-dependent: GRA+ diet led to the lowest rumen true N degradability, the highest overflow of rumen by-pass protein (NANM-N) and ultimately tended to have an increased flow of protein leaving the rumen as NAN. This observation could explain the greater growth rates described in lambs supplemented selenium and vitamin E (Naziroglu et al., 1997).

### Effect of forage type on the fermentation pattern

Successful haymaking relies on the grass being thoroughly dried before it is stored. Under our experimental conditions, HAY had a slightly lower N content (−7.6%) and an increased NDF (+9.2%) and ADF content (+17%) than GRA. Nutrient loss throughout haymaking is mainly due to degradation of sugars due to plant respiration, oxidation of fatty acids (Huws et al., 2009) and loss of nutrient-rich leaves during raking (Pizarro and James, 1972). Our experiment showed that this process did not promote changes in the OM disappearance in the fermenters. These findings agree with previous reports which showed no differences in DM degradability between fresh, frozen and dried grass (Minson, 1990). Similar lack of differences in rumen OM for timothy pasture vs. timothy hay were reported using the *in situ* method (Petit and Tremblay, 1992) or duodenally-cannulated cows (Halmemies-Beauchet-Filleau et al., 2013). However, our experiment showed that HAY had a greater NDF (+12%) and ADF (+26%) disappearance than GRA diets. This increased fiber disappearance in HAY diets may be due to the higher concentration of rumen protozoa (+0.25 log units) in vessels fed HAY than GRA, particularly those with a high fibrolytic activity such as the subfamily *Entodiniinae* (Dehority, 2003).

There is a general lack of consensus on the effect of the forage conservation method on the fermentation rate: it has been suggested that hay can increase (Mohammed et al., 2014), maintain (Halmemies-Beauchet-Filleau et al., 2013) or decrease (Holden et al., 1994) total VFA production with respect to the fresh pasture. However, it seems that in these studies VFA production was mainly driven by the feed DM intake which could explain the lack of differences across diets observed in our experiment. In the present study, a greater proportion of acetate and propionate, as well as a greater propionate outflow (+15%) were observed with HAY diets. On the contrary, GRA diets promoted a more acidic fermentation characterized by a lower pH and a greater lactate concentration and D/L lactate ratio. Both D and L isomers of lactate are produced in the rumen: in the presence of soluble sugars most D-lactate, and some L-lactate, are metabolized into propionate as the main product, whereas in the absence of soluble sugars only a small proportion of lactate is fermented to VFA, with acetate as the main end-product (Counotte et al., 1983). Moreover, it has been suggested that the activity of the principal enzymes involved in the metabolism of D-lactate decline at low rumen pH, resulting in an increased ratio of D/L lactate as more concentrate is added into the diet (Nocek, 1997). Petit and Tremblay (1992) noted that fresh grass had a greater soluble DM content but hay had a higher DM disappearance rate using the *in situ* technique. This observation is in line with our findings, since low pH in the vessels, together with higher lactate concentration (mainly as D-lactate) and higher levels of *Lactobacillus* (+38%), a lactate producing bacterium, seems to suggest that a greater availability of easily fermentable carbohydrates occurred with GRA in comparison to HAY diets. On the contrary, lower concentrations of lactate (mainly as L-lactate) together with the high levels of lactate utilizers such as *Selenomonas* (+94%) may suggest that most of the lactate was transformed into propionate with HAY diets. Moreover, CCA revealed that changes in the structure of the bacterial community induced by GRA were positively correlated with various parameters involved in the degradation of structural carbohydrates, such as fiber degradability and production of metabolic H, VFA and methane. This scenario may explain the higher acetate concentration observed in HAY in comparison with GRA diets because a positive correlation has been described between NDF content and acetate production (Nozière et al., 2011). Therefore, differences in the VFA proportions observed between GRA and HAY diets may be related to differences in the availability of specific nutrients (non-structural vs. structural carbohydrates) or in feed degradation kinetics (Petit and Tremblay, 1992) which ultimately determine the active metabolic pathways in the different microbes.

### Effect of forage type on rumen microbial communities

Although the forage conservation method had a minor effect on its chemical composition, it had a profound impact on the structure and diversity of the main microbial communities in the rumen. Thus, there may have been subtle differences with respect to physiological responses to plant stresses which can affect the rumen microbiome (Kingston-Smith et al., 2008). The lower bacterial concentration observed in HAY in comparison to GRA may indicate that less nutrients or growth factors are available in the former forage. This situation may have also been magnified by the presence of high levels of protozoa in HAY diets which exert a direct substrate competition with bacteria as a result of their ability to engulf large amounts of easily fermentable carbohydrates (Dehority, 2003). Higher levels of rumen protozoa were also described in cows fed diets containing 60:40 forage-to-concentrate ratio in which the forage source was hay instead of timothy-fescue pastures (Halmemies-Beauchet-Filleau et al., 2013). However, greater protozoal numbers were reported in cows grazing orchardgrass alone vs. orchardgrass hay (Holden et al., 1994) or fresh perennial ryegrass alone vs. grass hay (Huws et al., 2009). Rumen protozoa require a combination of structural and non-structural carbohydrates to thrive (Williams and Coleman, 1992); a combination which can be achieved by feeding fresh grass alone or feeding hay supplemented with concentrates (as in our study). Low bacterial numbers have also been described in the presence of rumen protozoa as a result of direct bacterial predation (Newbold et al., 2015), with the subfamily *Entodiniinae* particularly active to this respect (Belanche et al., 2012a). As a result of this processes, it has been reported that rumen protozoa are able to modify the structure of the bacterial commuity in the rumen (Belanche et al., 2012b, 2015b) and tend to increase bacterial diveristy (Newbold et al., 2015), as was noted in this experiment.

Fiber degradation is a complex process which requires a multitude of microorganism to work together (McAllister et al., 1994), thus a higher bacterial diversity has been observed in cows fed a fibrous diet (Belanche et al., 2012c). CCA revealed that the structure of the bacterial community in vessels fed HAY was highly correlated to the fiber degradation proceses (i.e., NDF disappearence, VFA, H and methane production and *Entodinium*). This adaptation to fiber utilization with HAY diets was associated to a greater complexity of the bacterial community in comparison with GRA diets (+38 OTUs); this community was dominated by the phylum Firmicutes (+20%) and also had increased levels of a combination of cellulolytic (*Ruminococcu*s), pectinolytic (*Treponema*), proteolytic (*Clostridium* and *Coriobacterineae*), amyolytic (*Mitsuokella* and *Proteobacteria*) and lactate producers (*Lachnobacterium*) and utilizers (*Selenomonas*). These bacterial species, togeher with protozoa, represents primary and secondary plant colonizers and could explain the greater fiber degradability with HAY diets (Edwards et al., 2007; Huws et al., 2016). On the contrary, GRA diets promoted a simplified bacterial community dominated by Bacteroidetes (+22% higher than with HAY diets) and a combination of hemicelulolytic (*Prevotella* and *Eubacterium*), amyolytic (*Syntrococcus*), lactate producers (*Lactobacillus*), and lypolytic bacteria (*Anaerovibrio*). A similar shift from Firmicutes toward Bacteroidetes has been described during the transition from forage to concentrate diets in cattle (Fernando et al., 2010) and sugests that more easily fermentable carbohydrates were available in GRA than in HAY diets.

Although the increase in total gas production (+12%) with HAY diets was of the same order of magnitude to the increase observed for fiber degradability, methane emissions increased to a greater extent per day (+35%), per degradable OM (+29%) and per unit of VFA (+23%), suggesting that HAY diets encourage methanogenic processes in the rumen. Methanogenic archaea are the sole producers of methane in the rumen (Morgavi et al., 2010), therefore a correlation between methanogens and methanogenesis might be expected (Wallace et al., 2014). Alternatively, a shift in the methanogen community toward one less effective in producing methane has also been sugested to explain rumen methanogenesis (Hegarty, 1999). Our qPCR analysis showed simililar methanogen levels across diets; however 16S amplicon sequencing revealed a slight change in the structure of the methanogen community. This shift was acompanied by an increased methanogen diversity in HAY diets and greater abundances of certain methanogen species in comparison to GRA diets (*Methanobacterium, Methanimicrococcus blatticola*, and *Methanobrevibacter ruminantium*). Therfore, the second hypothesis may be true under our experimental conditions and seems to rely on the different methanogenic potential observed among various methanogen groups (Hook et al., 2010). Our results showed that the metabolic H production was only slightly higher with HAY in comparison to GRA diets; however a much greater H incorporation (+23%) occurred with HAY than with GRA diets. As a result, most of the metabolic H (94%) was directed toward methane in HAY diets suggesting that an efficient inter-species H transfer between protozoa and methanogens occurred (Ushida et al., 1997). On the contrary, a substantial proportion of H (15%) was directed to alternative hydrogen sinks with GRA diets (mainly lactate). A number of mechanisms by which protozoa could enhance methanogenesis are possible since protozoa produce metabolic H, serve as host for methanogens and also protect them from oxygen toxicity (Belanche et al., 2014). CCA revealed that the structure of the methanogen community in vessels fed HAY diets was correlated with the presence of Holotrich and *Diplodiniinae* protozoa and with the methanogen diversity. These findings are in line with our previous observation which revealed that Holotrich protozoa have a predominant role in rumen methanogenesis (Belanche et al., 2015b) and have a community of endosymbiotic methanogens which differs from those found in other protozoal groups (Belanche et al., 2014). It has already been reported that dairy cows fed a total mixed diet produced more methane per unit of milk (+15%) than those grazing perennial ryegrass (O'Neill et al., 2011). Our experiment indicates that GRA diets produce less methane per unit of VFA or degradable OM than those based on HAY; therefore pasture base systems should be energetically more efficient than those based on hay diets and thus considered as a methane mitigation strategy.

### Effect of forage type on rumen N metabolism

An apparent inconsistency was noted in this experiment as the greater true rumen N degradability observed in HAY diets was accompanied with a lower ammonia concentration (+20%) and ammonia overflow (+23%) in comparison to GRA diets. Branched chain fatty acids result from the deamination of amino acids in the rumen (Firkins, 1996). Thus, the greater concentration of branched-chain fatty acids detected in vessels fed HAY (+5%) was consistent with the greater true N degradability (+7%). As a result of this feed proteolysis, HAY diets had a lower overflow of NANM-N (−31%), fraction which represents the rumen by-pass protein and is often needed to meet metabolizable protein requirements in high yielding cows (Firkins, 1996). Rumen ammonia concentration is not only affected by proteolysis, but also by ammonia uptake by the microbes. Thus, increased ammonia uptake by bacteria (+25%) may have contributed to lower ammonia levels with HAY diets. On the contrary, the greater levels of ammonia in GRA diets could be due to its greater N content (+6% than HAY) and to a greater concentration of *Prevotella* (+45%) which is considered the main microbe involved in the degradation of dipeptides into amino acids and ammonia (Wallace, 1996). In a compilation of various studies Minson (1990) also noted a higher NAN leaving the rumen (+9%) for fresh ryegrass than for dried ryegrass mainly as a result of its higher N content. However, the observed increased true N degradability in HAY diets disagrees with most of the literature (Minson, 1990; Holden et al., 1994); thus more measurements of the feed nutritional value such as *in situ* incubations, buffer soluble N and acid-detergent insoluble N should be used in future experiments to better understand the kinetic of nutrient degradation in these forages.

In previous *in vivo* (Belanche et al., 2012b) and *in vitro* (Belanche et al., 2016b) experiments we noted that a simplification in the complexity of the bacterial community is generally associated with a greater efficiency of N utilization but low fiber degradation. This observation was also true in this experiment where GRA diets, in comparison to HAY diets, had a positive impact on the microbial protein synthesis (+16%) and on the efficiency of microbial synthesis per unit of degradable OM (+21%) or rumen truly digested N (+18%). These observations were supported by the CCA which showed a positive correlation between the structure of the bacterial community in GRA diets and the microbial N synthesis and ultimately with the efficiency of feed utilization by the rumen microbes.

## Implications

This study demonstrated that a multi-omics approach based on a detailed characterization of the rumen microbiome coupled with an integrated description of the rumen fermentation pattern and N metabolism is vital to understand the effect and mode of action of different nutritional strategies. It was observed that forage conservation method (grass vs. hay) had a profound impact on the structure and diversity of the main microbial communities in the rumen (i.e., bacterial, protozoal, and methanogen communities). Hay diets promoted an increase in fiber and protein degradation but also in methane emissions (+35%), which may be associated with the greater protozoal concentration observed, as well as to the more diverse methanogen and bacterial community dominated by Firmicutes. On the contrary, grass promoted a more simplified structure and diversity in the rumen microbiome. The bacterial community was dominated by Bacteroidetes and had a greater bacterial growth and microbial protein synthesis (+16%) than described for hay diets. This study also revealed that the inclusion of vitamin E improved the rumen function. This improvement consisted of a small increase in feed degradability (+8%) which was associated with greater bacterial and protozoal levels. Our findings suggest that, in comparison to hay, grass feeding would lead to improved rumen function and should decrease the environmental impact of livestock agriculture. Further *in vivo* studies should be conducted using similar multi-omics approaches to better understand the interaction between forage, rumen function and microbiome on farm conditions.

## Author contributions

AB, AK, and CN designed the experiment; AB conducted the research and wrote the manuscript; AB, AK, and CN reviewed the manuscript. AB had primary responsibility for the final content. All authors read and approved the final manuscript.

### Conflict of interest statement

The authors declare that the research was conducted in the absence of any commercial or financial relationships that could be construed as a potential conflict of interest.
