# Supplementary material for: An Integrated Multi-Omics Approach Reveals the Effects of Supplementing Grass or Grass Hay with Vitamin E on the Rumen Microbiome and Its Function
Source: Front Microbiol. 2016 Jun 10;7:905. doi: 10.3389/fmicb.2016.00905 (PMC4901035; doi:10.3389/fmicb.2016.00905)
Supplement: Supplementary file 1 [file DataSheet1.docx]

**SUPLEMENTARY INFORMATION**

**Supplementary Table 1**. Primers used for quantitative PCR and Ion-Torrent Next Generation Sequencing.

| **Target** | **Author** | **Forward Primer** | **Reverse Primer** | **T^a^** | | **Amplicon (bp)** |
| --- | --- | --- | --- | --- | --- | --- |
| **Quantitative PCR** |  |  |  | |  |  |
| Total bacteria | ([Maeda*, et al.*, 2003](#_ENREF_42)) | GTGSTGCAYGGYTGTCGTCA | ACGTCRTCCMCACCTTCCTC | | 61 | 150 |
| Anaerobic fungi | ([Denman & McSweeney, 2006](#_ENREF_20)) | GAGGAAGTAAAAGTCGTAACAAGGTTTC | CAAATTCACAAAGGGTAGGATGATT | | 62 | 120 |
| Methanogens | ([Denman*, et al.*, 2007](#_ENREF_21)) | TTCGGTGGATCDCARAGRGC | GBARGTCGWAWCCGTAGAATCC | | 56 | 140 |
| **Ion Torrent NGS** |  |  |  | |  |  |
| Bacterial primers | ([Spear*, et al.*, 2008](#_ENREF_71)) | AGAGTTTGATCMTGGCTCAG | CTGCTGCCTYCCGTA | | 58 | 348 |
| Bacterial Adaptors |  | CCATCTCATCCCTGCGTGTCTCCGACTCAG | CCTCTCTATGGGCAGTCGGTGAT | |  |  |
| Methanogens primers | ([Wright & Pimm, 2003](#_ENREF_85)) | GCTCAGTAACACGTGG | GWATTACCGCGGCKGCTG | | 58 | 433 |
| Methanogens adaptors |  | CCATCTCATCCCTGCGTGTCTCCGACTCAG | CCTCTCTATGGGCAGTCGGTGAT | |  |  |

Denman SE & McSweeney CS (2006) Development of a real-time PCR assay for monitoring anaerobic fungal and cellulolytic bacterial populations within the rumen. *FEMS Microbiol. Ecol.* **58**: 572-582.

Denman SE, Tomkins N & McSweeney CS (2007) Quantitation and diversity analysis of ruminal methanogenic populations in response to the antimethanogenic compound bromochloromethane. *FEMS Microbiol. Ecol***. 62**: 313-322.

Maeda H, Fujimoto C, Haruki Y*, et al.* (2003) Quantitative real-time PCR using TaqMan and SYBR Green for *Actinobacillus actinomycetemcomitans , Porphyromonas gingivalis , Prevotella intermedia* , tetQ gene and total bacteria. *FEMS Immunol. Med. Microbiol.* **39**: 81 – 86.

Spear GT, Sikaroodi M, Zariffard MR, Landay AL, French AL & Gillevet PM (2008) Comparison of the diversity of the vaginal microbiota in HIV-infected and HIV-uninfected women with or without bacterial vaginosis. *J. Infec. Dis.* **198**: 1131-1140.

Wright ADG & Pimm C (2003) Improved strategy for presumptive identification of methanogens using 16S riboprinting. *J. Microbiol.l Meth.* **55**: 337-349.

**Supplementary Table 2.** Effect of the type of forage and vitamin E supplementation on the relative abundance of the main bacteria at phyla, families and genera in the Rusitec system.

|  |  |  | **Grass** | | **Grass hay** | |  | **Adjusted *P*-value** | | |
| --- | --- | --- | --- | --- | --- | --- | --- | --- | --- | --- |
| **Phylum** | **Family** | **Genus** | **GRA-** | **GRA+** | **HAY-** | **HAY+** | **SED^1^** | **F** | **V** | **F×V** |
| Actinobacteria | Coriobacteriales | Coriobacterineae | 1.53 | 1.50 | 1.70 | 1.77 | 0.123 | 0.030 | 0.981 | 0.891 |
| Bacteroidetes | |  | 3.70 | 3.72 | 3.65 | 3.60 | 0.022 | <0.001 | 0.66 | 0.195 |
|  | Bacteroidaceae | Bacteroides | 1.11 | 1.19 | 0.98 | 1.08 | 0.14 | 0.257 | 0.663 | 0.999 |
|  | Bacteroidales sedis | Phocaeicola | 1.68 | 1.63 | 1.67 | 1.48 | 0.101 | 0.324 | 0.305 | 0.725 |
|  | Cyclobacteriaceae |  | 0.63 | 0.69 | 1.18 | 1.06 | 0.183 | 0.005 | 0.981 | 0.861 |
|  | Marinilabiliaceae |  | 2.14 | 1.89 | 1.58 | 1.10 | 0.106 | <0.001 | 0.003 | 0.599 |
|  |  | Alkalitalea | 1.91 | 1.63 | 1.45 | 0.98 | 0.142 | <0.001 | 0.016 | 0.725 |
|  | Porphyromonadaceae | | 2.22 | 2.17 | 2.44 | 2.42 | 0.074 | <0.001 | 0.73 | 0.998 |
|  | Prevotellaceae |  | 3.42 | 3.47 | 3.31 | 3.33 | 0.036 | <0.001 | 0.305 | 0.843 |
|  |  | Hallella | 0.88 | 0.97 | 1.63 | 1.27 | 0.215 | 0.005 | 0.67 | 0.599 |
|  |  | Prevotella | 3.40 | 3.46 | 3.25 | 3.29 | 0.034 | <0.001 | 0.289 | 0.934 |
|  | Rikenellaceae | Rikenella | 1.13 | 1.15 | 1.46 | 1.22 | 0.091 | 0.009 | 0.305 | 0.295 |
|  | Sphingobacteriaceae |  | 1.90 | 1.97 | 2.08 | 1.70 | 0.113 | 0.670 | 0.296 | 0.155 |
| Elusimicrobia | |  | 1.66 | 1.45 | 1.83 | 1.63 | 0.081 | 0.010 | 0.016 | 0.999 |
|  | Elusimicrobiaceae | Elusimicrobium | 1.52 | 1.37 | 1.70 | 1.57 | 0.08 | 0.005 | 0.163 | 0.991 |
| Fibrobacteres | Fibrobacteraceae | Fibrobacter | 2.65 | 2.73 | 2.74 | 2.59 | 0.082 | 0.76 | 0.768 | 0.295 |
| Firmicutes |  |  | 3.58 | 3.55 | 3.61 | 3.68 | 0.025 | <0.001 | 0.498 | 0.174 |
|  | Acidaminococcaceae |  | 2.46 | 2.26 | 2.55 | 2.41 | 0.094 | 0.113 | 0.142 | 0.934 |
|  |  | Acidaminococcus | 1.46 | 1.26 | 1.54 | 1.19 | 0.099 | 0.884 | 0.003 | 0.725 |
|  |  | Succiniclasticum | 2.40 | 2.19 | 2.50 | 2.38 | 0.101 | 0.085 | 0.192 | 0.891 |
|  | Clostridiaceae 1 | Clostridium sensu stricto | 1.94 | 1.62 | 1.71 | 2.96 | 0.368 | 0.063 | 0.296 | 0.145 |
|  | Clostridiales Sedis XIII | | 1.95 | 1.84 | 2.06 | 2.02 | 0.068 | 0.010 | 0.305 | 0.861 |
|  |  | Anaerovorax | 1.90 | 1.80 | 2.01 | 1.95 | 0.066 | 0.018 | 0.305 | 0.934 |
|  |  | Mogibacterium | 0.90 | 0.77 | 1.07 | 1.16 | 0.157 | 0.034 | 0.981 | 0.725 |
|  | Erysipelotrichaceae |  | 2.02 | 1.90 | 1.80 | 1.76 | 0.072 | 0.005 | 0.342 | 0.808 |
|  | Eubacteriaceae |  | 1.16 | 1.02 | 0.62 | 0.68 | 0.155 | <0.001 | 0.905 | 0.725 |
|  | Lachnospiraceae |  | 2.76 | 2.73 | 2.73 | 2.69 | 0.049 | 0.310 | 0.645 | 0.999 |
|  |  | Butyrivibrio | 1.07 | 1.09 | 1.06 | 1.08 | 0.07 | 0.820 | 0.905 | 0.999 |
|  |  | Clostridium XlVa | 1.14 | 1.16 | 1.28 | 1.37 | 0.119 | 0.068 | 0.768 | 0.966 |
|  |  | Lachnobacterium | 1.08 | 1.11 | 1.33 | 1.29 | 0.058 | <0.001 | 0.981 | 0.808 |
|  |  | Lachnospiracea sedis | 1.61 | 1.64 | 1.56 | 1.47 | 0.081 | 0.092 | 0.802 | 0.725 |
|  |  | Oribacterium | 1.86 | 1.92 | 1.65 | 1.67 | 0.07 | <0.001 | 0.663 | 0.991 |
|  |  | Pseudobutyrivibrio | 1.61 | 1.56 | 1.45 | 1.57 | 0.057 | 0.092 | 0.66 | 0.295 |
|  |  | Roseburia | 1.67 | 1.73 | 1.51 | 1.59 | 0.125 | 0.128 | 0.73 | 0.999 |
|  |  | Syntrophococcus | 1.26 | 1.36 | 1.02 | 0.94 | 0.096 | <0.001 | 0.981 | 0.636 |
|  | Lactobacillaceae | Lactobacillus | 2.06 | 2.17 | 1.68 | 1.57 | 0.191 | 0.003 | 0.989 | 0.808 |
|  | Ruminococcaceae |  | 2.79 | 2.73 | 2.96 | 2.88 | 0.051 | <0.001 | 0.296 | 0.998 |
|  |  | Butyricicoccus | 1.27 | 1.09 | 1.74 | 1.33 | 0.16 | 0.009 | 0.142 | 0.725 |
|  |  | Clostridium IV | 1.03 | 0.87 | 1.25 | 1.11 | 0.116 | 0.018 | 0.296 | 0.999 |
|  |  | Ethanoligenens | 1.29 | 1.15 | 1.20 | 1.26 | 0.101 | 0.94 | 0.802 | 0.599 |
|  |  | Flavonifractor | 0.81 | 0.67 | 1.03 | 0.97 | 0.095 | <0.001 | 0.352 | 0.891 |
|  |  | Oscillibacter | 1.66 | 1.52 | 1.37 | 1.50 | 0.088 | 0.035 | 0.989 | 0.287 |
|  |  | Ruminococcus | 1.78 | 1.79 | 2.10 | 2.08 | 0.108 | <0.001 | 0.989 | 0.999 |
|  |  | Sporobacter | 1.02 | 0.93 | 1.24 | 1.02 | 0.093 | 0.04 | 0.165 | 0.725 |
|  | Streptococcaceae | Streptococcus | 2.53 | 2.52 | 2.34 | 2.33 | 0.213 | 0.257 | 0.989 | 0.999 |
|  | Veillonellaceae |  | 2.55 | 2.62 | 2.74 | 2.80 | 0.07 | <0.001 | 0.436 | 0.999 |
|  |  | Anaerovibrio | 1.72 | 1.83 | 1.57 | 1.68 | 0.07 | 0.012 | 0.242 | 0.999 |
|  |  | Mitsuokella | 1.74 | 1.74 | 2.09 | 2.07 | 0.13 | 0.003 | 0.989 | 0.999 |
|  |  | Schwartzia | 1.78 | 1.90 | 1.84 | 1.85 | 0.054 | 0.932 | 0.305 | 0.599 |
|  |  | Selenomonas | 2.09 | 2.18 | 2.38 | 2.43 | 0.08 | <0.001 | 0.402 | 0.991 |
| Proteobacteria | |  | 2.52 | 2.51 | 2.65 | 2.69 | 0.056 | <0.001 | 0.883 | 0.843 |
|  | Succinivibrionaceae | Succinivibrio | 1.53 | 1.57 | 1.63 | 1.70 | 0.102 | 0.122 | 0.738 | 0.999 |
| Spirochaetes | |  | 2.07 | 2.09 | 2.28 | 2.10 | 0.068 | 0.036 | 0.305 | 0.295 |
|  | Spirochaetaceae |  | 2.04 | 2.04 | 2.25 | 2.06 | 0.074 | 0.057 | 0.296 | 0.472 |
|  |  | Sphaerochaeta | 1.94 | 1.92 | 2.05 | 1.86 | 0.095 | 0.745 | 0.305 | 0.654 |
|  |  | Treponema | 1.20 | 1.27 | 1.49 | 1.43 | 0.088 | 0.003 | 0.989 | 0.725 |
| Tenericutes | Anaeroplasmataceae |  | 2.54 | 2.46 | 2.39 | 2.42 | 0.058 | 0.045 | 0.781 | 0.599 |
|  |  | Anaeroplasma | 2.50 | 2.43 | 2.37 | 2.40 | 0.059 | 0.099 | 0.802 | 0.725 |
|  |  | Asteroleplasma | 1.47 | 1.23 | 0.96 | 1.14 | 0.12 | 0.005 | 0.923 | 0.195 |
| Unclassified | |  | 2.32 | 2.32 | 2.52 | 2.32 | 0.048 | 0.010 | 0.087 | 0.145 |

^1^Standard error of the difference for the interaction between the type of forage and the vitamin E supplementation at 50 IU/d (F×V). The total number of reads per sample was log-transformed and minor genera (<0.1%) were discarded. Samples were taken at 4 and 24h after feeding (*n*=8). Within a row means without a common superscript differ (*P* < 0.05)

**Supplementary Table 3.** Effect of the type of forage and vitamin E supplementation on the relative abundance of the main methanogenic archaea at family, genus and specie level in the Rusitec system.

|  |  |  | **Grass** | | **Grass hay** | |  | ***P*-value** | | |
| --- | --- | --- | --- | --- | --- | --- | --- | --- | --- | --- |
| **Family** | **Genus** | **Specie** | **GRA-** | **GRA+** | **HAY-** | **HAY+** | **SED^1^** | **F** | **V** | **F×V** |
| *Methanobacteriaceae* |  |  | 2.12 | 1.98 | 2.13 | 2.05 | 0.084 | 0.541 | 0.080 | 0.591 |
|  | *Methanobacterium* |  | 0.25 | 0.23 | 0.59 | 0.39 | 0.139 | 0.016 | 0.267 | 0.370 |
|  | *Methanobrevibacter* |  | 2.12 | 1.98 | 2.12 | 2.04 | 0.084 | 0.610 | 0.082 | 0.579 |
|  |  | *M gottschalkii* | 2.10 | 1.96 | 2.10 | 2.02 | 0.085 | 0.636 | 0.090 | 0.594 |
|  |  | *M ruminantium* | 0.68 | 0.49 | 0.75 | 0.73 | 0.128 | 0.099 | 0.232 | 0.356 |
| *Methanomicrobiaceae* | *Methanomicrobium* | *M mobile* | 1.54 | 1.92 | 1.62 | 2.12 | 0.159 | 0.226 | <0.001 | 0.589 |
| *Methanosarcinaceae* | *Methanimicrococcus* | *M blatticola* | 0.88 | 0.68 | 1.22 | 0.98 | 0.128 | 0.002 | 0.020 | 0.844 |
| *Methanomassiliicoccaceae* | *Methanomassiliicoccus* | | 3.19 | 3.17 | 3.17 | 3.14 | 0.019 | 0.213 | 0.085 | 0.154 |
|  |  | *Group3* | 1.85 | 1.59 | 1.86 | 1.63 | 0.084 | 0.649 | <0.001 | 0.836 |
|  |  | *Group8* | 2.53 | 2.50 | 2.48 | 2.46 | 0.056 | 0.289 | 0.577 | 0.921 |
|  |  | *Group9* | 2.91 | 2.94 | 2.89 | 2.92 | 0.018 | 0.167 | 0.037 | 0.896 |
|  |  | *Group11* | 1.38 | 1.02 | 1.46 | 1.14 | 0.109 | 0.243 | <0.001 | 0.794 |
|  |  | *Group12* | 2.40 | 2.28 | 2.27 | 2.29 | 0.054 | 0.130 | 0.196 | 0.100 |

^1^Standard error of the difference for the interaction between the type of forage and the vitamin E supplementation at 50 IU/d (F×V). The total number of reads per sample was log-transformed and minor genera (<0.1%) were discarded. Samples were taken at 4 and 24h after feeding (*n*=8). Within a row means without a common superscript differ (*P* < 0.05)

**
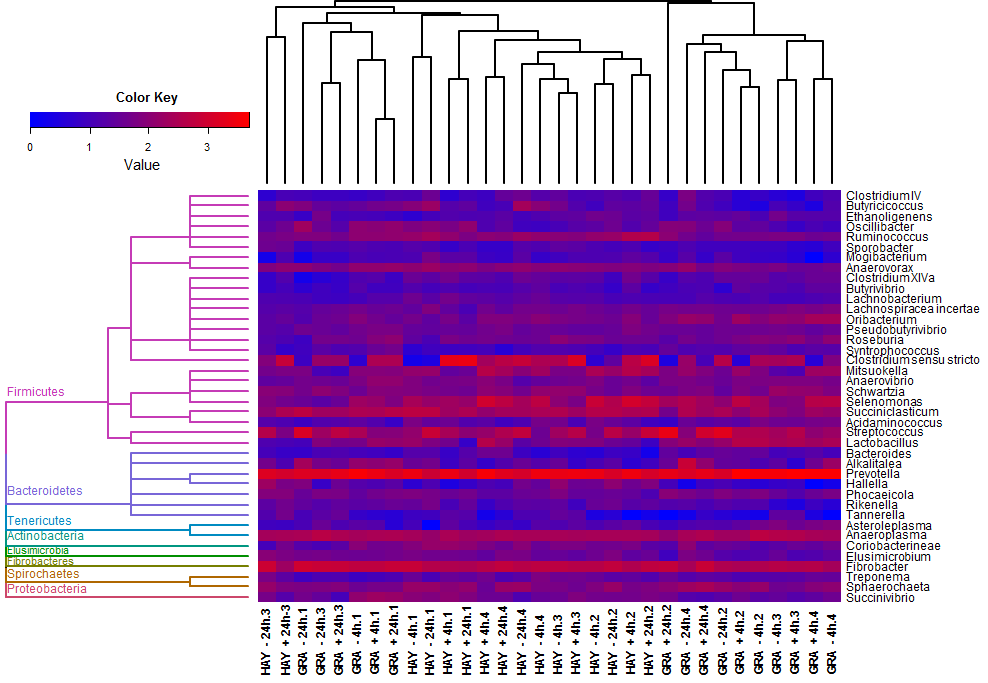
**

**Supplementary Figure 1.** Heat map describing the effect of grass (GRA), grass hay (HAY) and vitamin E supplementation (+ vs -) on the structure of the rumen bacterial community at the genus level in the Rusitec system. Dendrograms are based on the UPGMA clustering of the Bray-Curtis distances. The total number of reads per sample was log transformed and minor genera were discarded (<0.1%). Cows used as inoculum and sampling times are indicated in numbers.

**
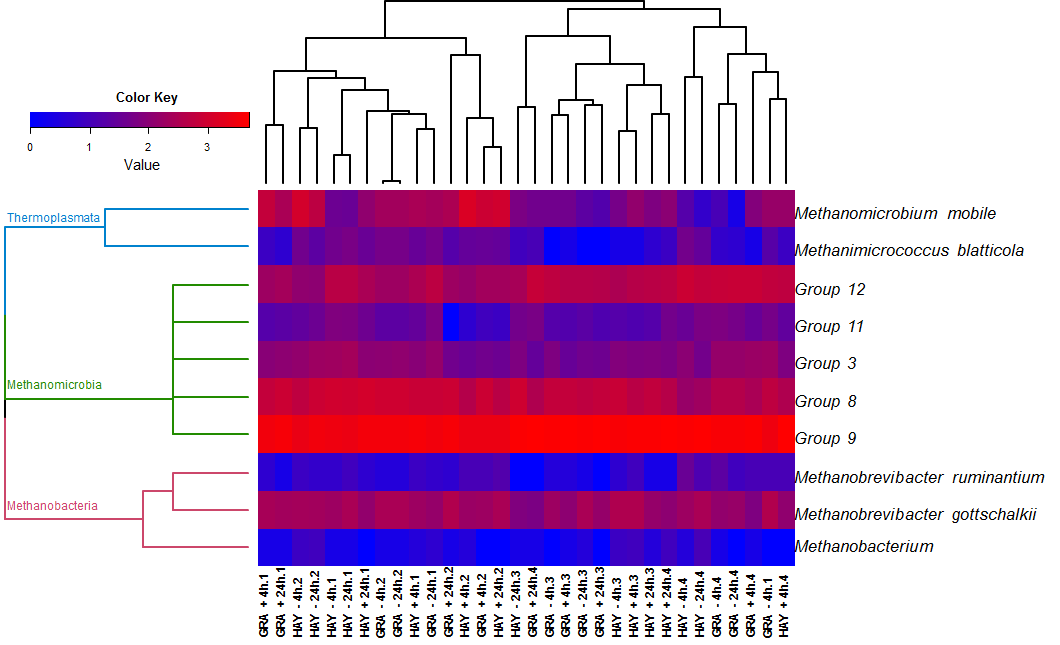
**

**Supplementary Figure 2.** Heat map describing the effect of grass (GRA), grass hay (HAY) and vitamin E supplementation (+ vs -) on the structure of the rumen methanogen community at the species level in the Rusitec system. Dendrograms are based on the UPGMA clustering of the Bray-Curtis distances. The total number of reads per sample was log transformed. Cows used as inoculum and sampling times are indicated in numbers.
